# Environmental stressors associated with suicidal behavior in adolescents with psychiatric pathology

**DOI:** 10.1192/j.eurpsy.2021.1701

**Published:** 2021-08-13

**Authors:** S. Pérez Sánchez, I. Martín Herrero, M.A. Cutillas Fernández, D. Raya Güimil, A. Crespo Portero

**Affiliations:** 1 Servicio De Psiquiatría, Servicio Murciano de Salud, Murcia, Spain; 2 Servicio De Psiquiatría, Servicio Murciano de Salud, Lorca, Murcia, Spain

**Keywords:** suicidal behavior, environmental stressors, adolescents, adverse life events

## Abstract

**Introduction:**

In the assessment of suicidal behavior, recent studies describe the great influence of an environmental component with adverse life events and stressors that can influence self-harm ideation and gesture.

**Objectives:**

1. To analyze the reasons for consultation of adolescents between 11 and 17 years of age who consult for suicidal behavior. 2. To estimate the frequency of the different socio-family life events.

**Methods:**

A retrospective review of the emergency room visits in the last 3 months was carried out. Sociodemographic data, vital events, reason for consultation and evolution are collected in the following 30 days after consultation in the emergency room.

**Results:**

Data were collected from 16 adolescents who consulted in the emergency room for suicidal ideation / gesture in a period of 3 months, of which 43% (7) were women and 56% (9) were men between 11 and 18 years old. The reasons recorded as stressful life events were: 22% unstructured family environment, 10% death of a close relative, 43% little parental supervision, 26% end of a romantic relationship, 5% legal problems, 2% sexual or physical abuse, 70 % academic problems, 3% bullying. It was observed that in 63% of the cases they presented more than one adverse experience.

**Conclusions:**

Suicidal ideation and behavior are frequently preceded by different adverse life events that can be minimized or go unnoticed and undervalued. A meticulous medical history can clarify some of the reasons that influence the hopelessness and clinical anguish that the suicidal patient presents. Its early detection provides the opportunity for an early and specialized approach.

**Disclosure:**

No significant relationships.

